# Impact of species composition on fire-induced stand damage in Spanish forests

**DOI:** 10.1038/s41598-024-59210-4

**Published:** 2024-04-13

**Authors:** Marina Peris-Llopis, Blas Mola-Yudego, Frank Berninger, Jordi Garcia-Gonzalo, José Ramón González-Olabarria

**Affiliations:** 1 School of Forest Sciences, University of Eastern Finland, Yliopistokatu 7, PO Box 111, 80101 Joensuu, Finland; 2 Department of Environmental and Biological Sciences, University of Eastern Finland, PL 1627, 80101 Joensuu, Finland; 3 Joint Research Unit CTFC – AGROTECNIO, Ctra de St. Llorenç de Morunys, Km 2, 25280 Solsona, Spain

**Keywords:** Environmental impact, Fire ecology, Forestry

## Abstract

Mixed forests play a fundamental ecological role increasing biodiversity and providing ecosystem services; it has been suggested they have higher resilience and resistance against disturbances, particularly fire. Here, we compare tree mortality in post-fire mixed and pure stands in Spain, on 2,782 plots and 30,239 trees during the period 1986 to 2007. We show evidence that mixed stands can have higher post-fire mortality than pure stands, and specific mixtures of species with different fire-related strategies increase the stand's vulnerability to fire damage versus pure stands of either species, such is the case of *Pinus halepensis*—*Pinus nigra* mixtures. Mixtures of two species often had higher mortality than species growing in pure stands. Combinations of species with different fire-related strategies can both enhance or reduce forest resistance. The role and management of mixed forests should be reconsidered after these findings, in order to enhance forest resilience to fires.

## Introduction

Mixed forests are widely considered as ecologically desirable as they provide a wide range of ecosystem services^1^ and particularly, the importance of tree species diversity to enhance resistance and resilience to disturbances is widely recognized and a topic of growing interest within the scientific community^2^. The relationships between species mixtures and the impact of disturbances have been studied, for example regarding resistance to drought or vulnerability to storm damage^3,4^. Concerning forest fires, however, the links between species mixtures and fire resistance have been more ambiguous.

Traditionally, most studies have focused on fire behaviour and tree level mortality on pure stands or single species (e.g., Alvarez et al.^5^; Fernandes et al.^6^): it is assumed that those forests are particularly vulnerable to fire damage due to their homogeneous composition (specially in even-aged stands and depending on management) and lower structural diversity compared to mixed forests, which can lead to higher levels of crown scorch during crown fires, causing significant mortality and reduced growth in the post-fire period. Mixed forests, on the other hand, can present more complex stand structures due to the higher diversity of species, resulting in a wider range of fire-related traits, among others: the ability to resprout after a fire facilitating post-fire regeneration^7,8^ or a thick bark protecting the tree sensitive tissues from heat, favouring its survival in case of surface fire^9,10^. Studies have shown that on regions where recurrent fires are common, mixed stands sustain less intense fires than pure coniferous forests^11,12^, resulting in lower extent fires^13,14^, lower severity and subsequent smaller damages^15^.

The implications of diverse species mixtures on post-fire mortality can, however, be diverse, as species have different morphological traits as a result of the fire related strategies of plants, on their co-evolution with fire regimes^16^. Additionally, mixed stands can present different fire behaviours (influenced by stand structure and fuel accumulation), leading to differences on fire severity^17^. These strategies can be defined as: fire-avoider species, species in closed canopy forests with limited fire tolerance that rely on high moisture values in order to generate a near-zero fire environment^18,19^, fire-resistant species, which are adapted to recurrent surface fires that periodically clear the surface fuels^9^ and fire-resilient species, which aim to colonise and persist in an area after a fire (whether by resprouting or through seedling recruitment), thus relying for their success on the occurrence of high intensity fires, favoured through increased flammability^20–22^. The combination of these strategies on mixed forests can question the idea that pure forests are necessarily subject to higher fire damage. There is evidence that pure broadleaved forests are less prone to experience high probability of fire occurrence and are more fire resistant than mixed stands of broadleaves and other species composition^23,24^. However, there is a limited number of studies concerning the influence of species mixtures on fire behaviour and resistance based on empirical data^25^.

Here, we assess post-fire stand damage in pure and mixed stands in Spain based on extensive data from the national forest inventory, during the period 1986 to 2007. For this purpose, we select plots in burnt areas representing different species mixtures, with a gradient of species composition, as well as pure stands. On these plots, we fit a general damage model using variables describing stand structure, and we assess the deviations in the predictions according to the species composition. The resulting values are analysed according to fire-related strategies based on the species traits. The study aims to understand the differential post-fire damage linked to the species composition with an empirical basis, and to assess the overall forest vulnerability to fire damage, particularly in Mediterranean areas.

## Results

**Figure 1:**
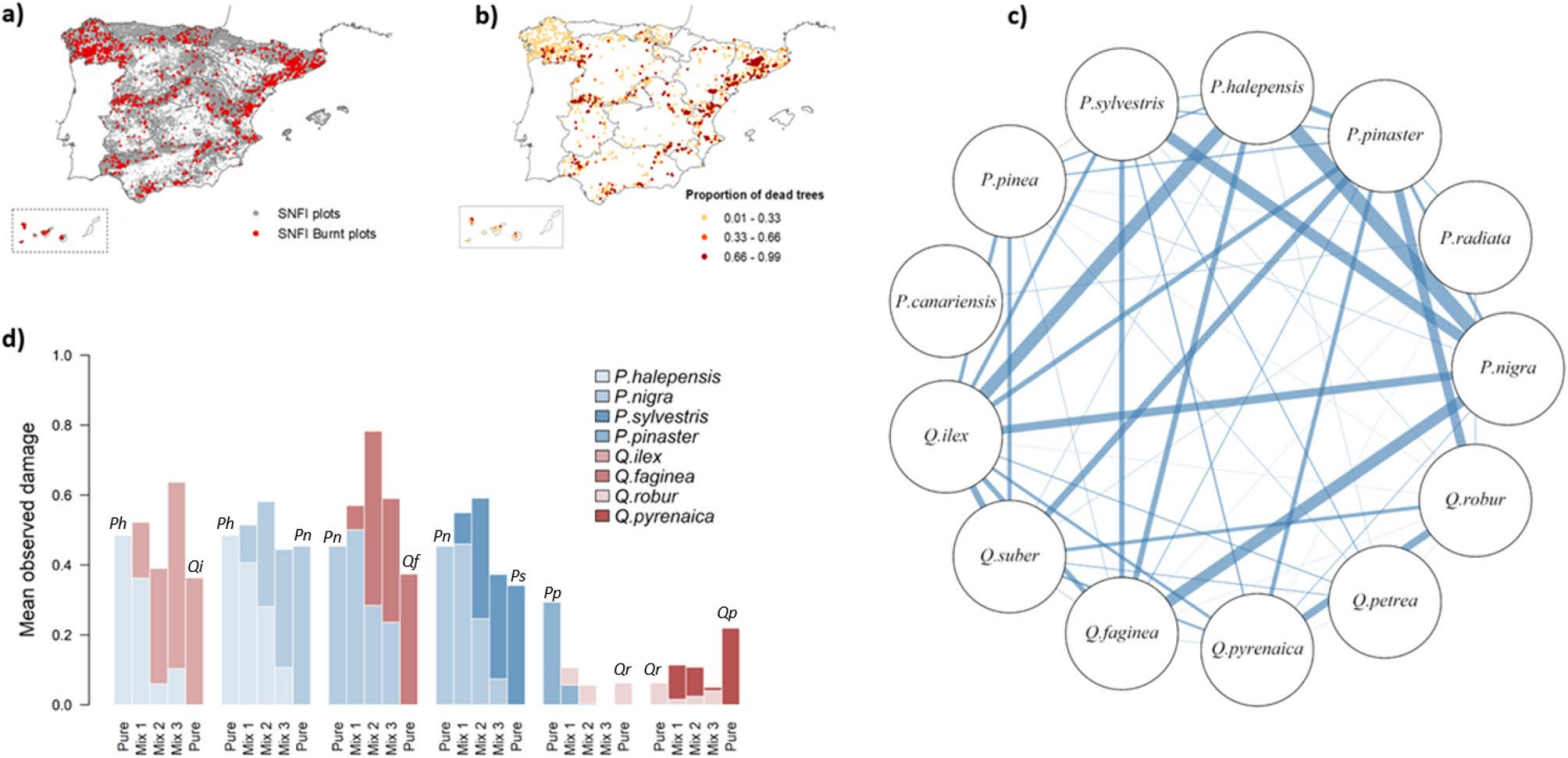
**(a, b)** Spanish National Forest Inventory plots and fire damage**.** Observed proportion of dead trees in the burnt plots**.** (**c**) Most common tree species and mixtures in the burnt plots. Line width represents the number of species co-occurrences. (**d**) Fire damage by species and their mixtures. Mean observed damage by species in the inventory plots affected by fire, in pure plots and according to three mixture levels (mix 1: 75%, mix 2: 50% and mix 3: 25%). Species initials indicate the main species in pure plots, with a left-to-right decreasing gradient in their proportion in the mixtures.

The analysis of the Spanish National Forest Inventory (NFI) data resulted in 2 782 plots and 30 239 trees affected by fire between 1997 and 2007 (Fig. 1a). Fire occurrence was unevenly distributed across the territory, with evident spatial aggregations: the highest proportion of burnt trees was mostly observed in Mediterranean regions in Eastern Spain (Fig. 1b), whereas other regions presented lower fire damage. These areas presented point aggregations with high damage values, which in many cases could be attributed to a single fire event. Plots with superficial fire damage (< 33% of fire damage, namely percentage of dead trees due to fire in the plot) or nearly completely burnt (fire damage higher than 66%) were more frequent than plots with intermediate post-fire mortality levels.

### Fire damage model

The developed model accounts for fire damage at stand level, focusing on the effects of stand structural characteristics and site conditions (Eq. 1). This model considers the effect of stand structure on the degree of fire damage, allowing to separate it from the effect of species composition and mixtures. According to our results (Table 1), stand basal area (*G*) negatively affects the proportion of dead trees in the stand, whereas the interaction *G* x *Dq*^*−1*^, which is non-linearly related to the number of trees per hectare, positively influences the proportion of dead trees by fire, showing that denser stands, characterised by larger values of *G* x *Dq*^*−1*^, present higher fire damage. In relation to site conditions, stands located in steeper slopes presented a higher fire related mortality. Mean values of the modelling variables in the selected mixtures are available as supplementary material (Table 1A).

**Table 1:** Influence of stand structure and site conditions on fire damage.

	Variable	Coefficient	SE
β_0_	Intercept	− 0.833	0.169
β_1_	G	− 0.182	0.010
β_2_	Slope (3–12%)	0.561	0.196
β_3_	Slope (12–20%)	0.384	0.184
β_4_	Slope (20–35%)	0.562	0.174
β_5_	Slope (> 35%)	0.983	0.172
β_6_	G x Dq^*−*1^	2.231	0.141

Regression coefficients of the stand level damage model.

All the coefficients were significant (*p*-value ≤ 0.05).

For the stand damage model, McFadden's pseudo-R^2^ was 0.13 and Nagelkerke pseudo-R^2^ was 0.21. The model residuals (observed minus predicted) ranged from − 0.65 to 0.97, and the absolute bias was − 0.037 (a slight over-estimation of fire damage). The model assumed the residuals to follow a quasibinomial distribution, which had more frequent negative values. Variance Inflation Factors smaller than 5 indicated no strong correlation between the modelling variables.

### Stand damage on mixed forests

Among the burnt forests, monospecific plots were particularly represented by *Pinus* species (728 burnt plots), followed by *Quercus* species (242 plots). Mixed species forests included 1 812 plots, with at least more than one species mixed at different proportions (Fig. 1c and 1d). Among those, the most frequent combinations, in decreasing order, were *Pinus nigra—Pinus halepensis*, *Quercus ilex—P. halepensis, P. nigra*—*Quercus faginea*, *Pinus sylvestris—P. nigra, Quercus robur—Pinus pinaster* and *Q. robur—Quercus pyrenaica. Pinus canariensis*, a species mainly present in the Canary Islands, was the main species in 440 burnt plots. In those, the observed damage was quite low, with a mean proportion of dead trees around 4%. Other species present in the burnt plots, but rarely mixed or mixed at low proportions, were: *Castanea sativa* (in pure stands or mixed with *Quercus suber*), *Fagus sylvatica* (pure and mixed, mostly with *Quercus petraea*), *Eucalyptus globulus* (mostly in pure stands) and *Olea europaea* (mixed with *P. halepensis*). In this regard, 41 species were present in less than 20% of the burnt plots and the remaining species were mostly *Pinus* and *Quercus*.

In mixed plots, the mean observed damage varies greatly according to changes in species composition and mixture levels (Fig. 1d), being higher at maximum mixture levels in half of the analysed mixtures when compared to pure plots of the same species. On the other hand, two mixtures showed lower damage at different mixture proportions: *P. pinaster* with *Q. robur* and *Q. pyrenaica* with *Q. robur*. In the case of *P. halepensis* and *Q. ilex* plots, mean observed damage is lower at maximum mixture levels compared to highly dominated *P. halepensis* stands, but increases in the other mixture levels. In general, when *Quercus* and *Pinus* species were mixed at similar proportions, *Quercus* shared a larger part of the fire-related mortality than pines.

**Figure 2:**
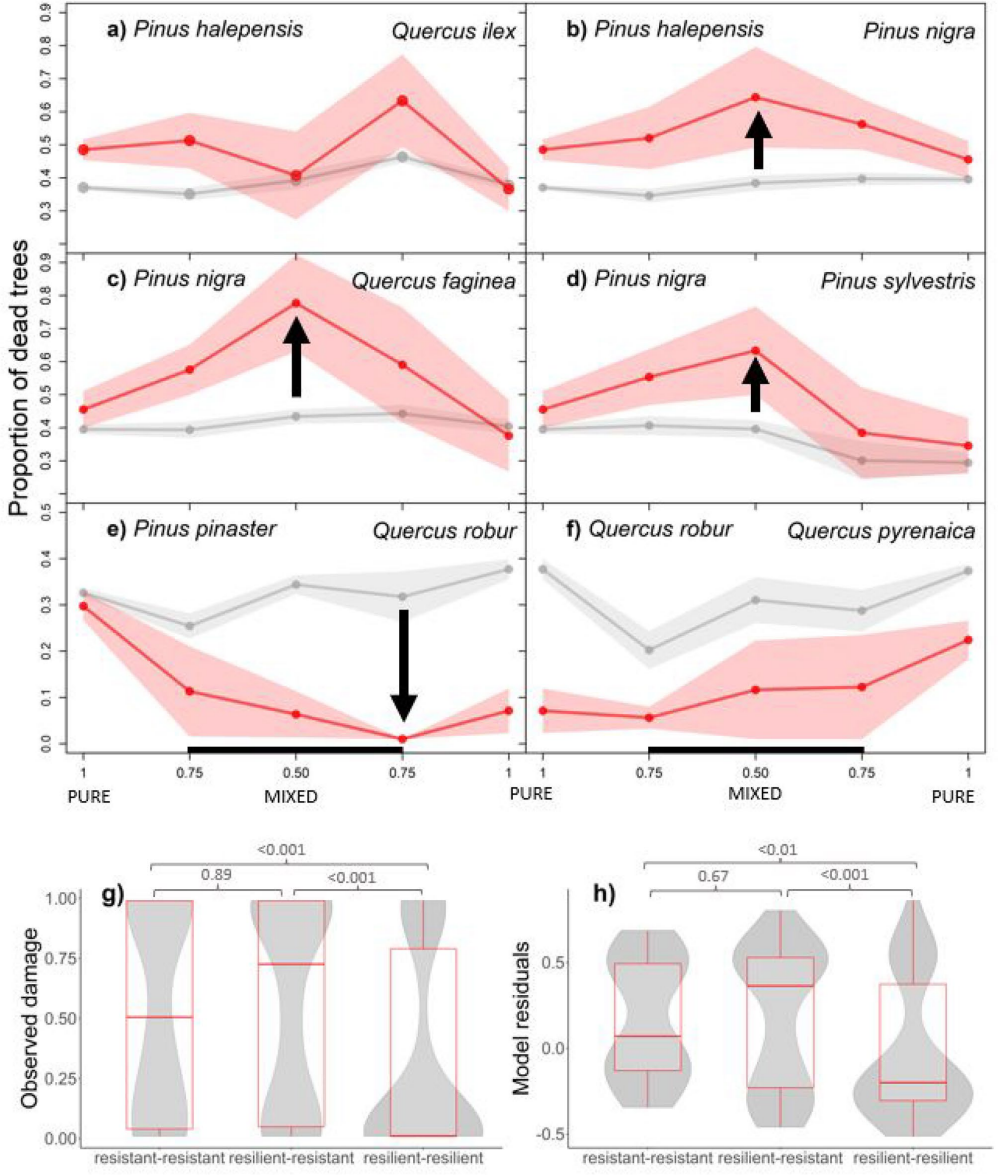
Observed and predicted fire damage by species mixtures. Mean predicted and observed fire damage from the model for the most common mixtures, based on the proportion of stand basal area occupied by each species. (**a–f)** modelled predictions (grey) versus observed values (red). The areas represent the mean’s standard error, and largest deviations between both are highlighted with arrows. (**g)** Distribution of observed stand damage, for the present combinations of fire-related strategies. (**h)** distribution of resulting model residuals for the present combinations of fire-related strategies. Post-hoc p-values for the between-groups comparisons are indicated on top of brackets connecting the compared groups.

The analysis of the model residuals, observed and predicted proportions of dead trees in the burnt plots reveals changes in fire damage in mixed plots compared to pure plots (Fig. 2). In the case of pure plots, observed damage was always lower in pure *Quercus* stands in comparison to pure *Pinus* stands. In mixed stands, the proportion of dead trees drastically varied depending on the proportion of stand basal area occupied by each species. For example, damage in mixed *P. nigra*—*Q. faginea* stands was higher when both species were present at similar proportions (50%). In this case, the mixture of species has a negative effect, as the mean observed damage is lower in pure plots and increases when both species are present. Overall, the mixtures of *P. nigra—P. halepensis*, *P. sylvestris—P. nigra,* and *P. nigra*—*Q. faginea* show negative effects on stand resistance to fire damage. The mixture of *Q. robur* with *P. pinaster*, mostly found in burnt plots in North-western Spain, did not show a clear effect of admixing on fire damage. The reduction in fire damage seems to be linked to the increasing occupation by *Q. robur.* In this regard, plots with these species mixed at a 75% level (N = 5) did not present fire damage. Similarly, the change in fire damage in the mixture *Q. robur*—*Q. pyrenaica* could be derived from the higher proportion of *Q. pyrenaica*, instead of showing a negative mixture effect.

The model residuals reflect how much the model predictions differ from observed values, with negative residuals indicating higher predicted damage than observed and, conversely, positive residuals indicate otherwise. In most of the mixtures, the model tends to underestimate damage, particularly when the proportions are close to 50–50. The exceptions are mixtures of *Q. robur—P. pinaster* and *Q. robur—Q. pyrenaica*, where results suggest a lower fire damage than the average predictions of the model according to their structure. In most of the pure stands, the model predictions were rather accurate, suggesting the structure variables included in the model accurately represent fire damage, regardless of the species composition. This is the case, as well, of one of the studied mixtures, *P. halepensis* with *Q. ilex*. In the case of stands of *Q. robur* and *Q. pyrenaica*, there was a consistent over-estimation of the damage, although there was no indication this ratio changed due to the proportion of species mixtures.

There are three fire related strategies combined in the analysed mixtures, fire-resistant species mixed with fire-resistant species (one mixture: *P. nigra*—*P. halepensis*), fire-resistant combined with fire-resilient species (one mixture; *P. nigra—Q. faginea*) and fire-resilient with fire-resilient species (four mixtures). Kruskal–Wallis test revealed significant differences in the observed proportion of dead trees or observed damage between the combinations of strategies (p-value < 0.001), in the predicted damage (p-value = 0.004) and in the model residuals (p-value < 0.001).

The distribution of observed damage (Fig. 2g) and model residuals (Fig. 2h) were connected to the fire-related strategies of the species mixtures. In mixed stands of species with a *fire-resistant* strategy, the observed damage was higher than in the stands classified as fire-resilient. In stands with both strategies simultaneously, the observed damage was the highest. Using the model to predict stand damage, the estimated mean residuals were 0.17, 0.16 and − 0.03 for fire-resistant, mixed resistant-resilient and fire-resilient stands, respectively, suggesting an underestimation of damage in the first two combinations and overestimation in stands with fire-resilient species.

Two-sided post-hoc Dunn's tests were applied to determine if there were significant differences between groups of strategies. Differences in the observed damage, predicted damage and model residuals were significant at a 0.05 alpha level for the following comparisons: resilient-resilient (N = 99) vs resilient-resistant (N = 100), and resilient-resilient vs resistant-resistant (N = 43). All p-values were significant when comparing resilient-resilient vs resilient-resistant plots (p < 0.001 for observed damage, p-value = 0.002 for predicted damage, and p-value < 0.001 for the model residuals). When comparing resilient-resilient vs resistant-resistant plots, results were: p-value < 0.001 for observed damage, p-value = 0.024 for predicted damage, and p-value = 0.003 for the residuals. There were no significant differences when comparing mixed stands of fire-resilient and fire-resistant species with mixed stands of fire-resistant species (observed damage p-value = 0.937, predicted damage p-value = 0.891, and residuals p-value = 0.678). These results highlight the differences on fire damage due to the combination of fire-related strategies and their traits.

## Discussion

The study addresses fire-induced stand damage in mixed and pure stands based on a large set of empirical measurements from diverse forest conditions. In Spain, pure and mixed forests include both Temperate and Mediterranean biomes and present a large tree species diversity due to different biogeographical factors. Spanish mixed forests entail about 27% of the Mediterranean natural forest area^26^, justifying their study as largely representative of forests subject to recurrent fires^27^. The importance of tree species diversity to enhance resistance and resilience to disturbances is a topic widely researched, with several studies^2–4^ showcasing the role of different species promoting forest resilience and the production of ecosystem services^1,25^. Specifically, the combination of distinct species groups, such as oaks versus pines, is claimed to enhance fire resistance^25^; however, the precise contribution of individual species and their combination levels remains unresolved.

We produced a general damage model that could be applied to the whole country, since up to date there are no tools based on empirical observations in this direction. Stands located in steeper slopes present higher proportions of dead trees than those in flatter locations, partly due to limited growth and tree vigour due to poor site conditions (thinner soils, leaching of nutrients, erosion, etc.), as well as a result of faster fire spread, higher flame length and intensity. At the same time, these locations present conditions that favour larger burnt areas, for example less accessibility and larger forest cover^28^. Regarding stand structure, both stand basal area and its ratio with mean quadratic diameter (*G x Dq*^*−1*^) had a strong influence on post-fire stand damage, with higher proportions of dead trees in denser stands, increasing the transmission of fire between trees. The overall results and the effects of the variables were consistent with previous studies with a similar modelling approach^29–31^.

The model was instrumental to differentiate effects due to stand structure from those due to species composition, by using the residual variance to assess differential tree mortality in different proportions and combinations of the species mixtures. The approach is in line with e.g., Dobbertin^32^, using model residuals as indicators of the effect of environmental conditions on tree growth, or Díaz-Yáñez et al.^33^, addressing differential mortality in stands attributed to ash dieback. Similarly, Jonsson et al.^34^ compared modelling results and observed levels of ecosystem services in mixed vs pure forests, attributing the differences in mean values to the mixture effect. The results of fire damage in monospecific plots were in line with González et al.^35^, except in the case of pure *Q. faginea* and pure *Q. ilex* plots, where higher mortality was observed. This could be due to methodological differences in the definition of pure plots (i.e., we consider 100% basal area as a threshold, instead of 80%), the larger study area and the way in which fire occurrence was determined. In mixed plots, the observed damage varied greatly according to changes in the species composition and degree of mixture; reflecting a more complex pattern.

Mixed forests often present more complex structures as well as a broader variety of morphological traits, influencing the impact of fire^36,37^. In this way, mixtures of species with thick barks would present a lower stand damage if a fire occurs, as it is the case of the mixture *P. pinaster—Q. robur* with decreasing observed damage in the studied plots. Mixed forest stands present different size-structure dynamics than pure stands^38^, which can favour or hinder the horizontal and vertical spread of fire. Self-pruning of branches prevents surface fires from spreading to the canopy^39–41^ by favouring gaps in the stand vertical structure, a trait that prevents crown fires that can result in higher damage levels. This advantage is diminished when self-pruning species are mixed with species that do not present this trait, as seen in the *P. nigra—P. halepensis* plots, which showed higher damage values at intermediate mixture levels. This results in a higher likelihood of crown burning due to fire transmission to the upper tree layer, especially when species with large height differences are mixed. The presence of serotinous cones and seed banks in the canopy allow the post-fire reproduction of some pine species^42^. At experimental level, fuel flammability experiments have revealed non-additive effects of mixing species^36,43,44^, which suggest that the most flammable fuels will be the ones driving fire characteristics, even if there is evidence that fire mitigation effects can also be found^45^.

Interactions among species composition, stand structure, and site conditions collectively influence the distribution of fuel loads and their flammability, for example through variations in moisture content^45–47^. The presence of distinct layers within the vertical forest structure significantly impacts fire spread, with stands typically characterized by a cohesive understory and tree layer facilitating surface fire escalation to the crowns, potentially exacerbating damages. However, the study of this assumption is challenged by numerous interrelations and confounding factors. We added understory mean top height in the characterization of stand structure in the mixed plots (see Table A1), but we did not find a clear connection with higher fire damage, except in *Q. robur* and *Q. pyrenaica* mixtures. In this case, a larger fire damage seems to correlate with higher understory heights, which could help connect both vegetation layers. However, our posterior analyses did not find a relationship between the model residuals and the mean understory height, suggesting that this effect of the vegetation structure is already explained by the other variables in the analysis.

Contrary to the general idea (see Bauhus et al.^25^), our results demonstrate that species mixtures, in many cases, exacerbate fire damage. Our results show that stand resistance to fire damage varies with species and their relative abundance, resulting in higher proportions of dead trees in mixed stands in the majority of the studied mixtures. These findings, together with the combination of other fire related traits can explain the negative effects found in the studied mixtures, always considering the role of fuel structure in the spread of fire^48^ and associated tree mortality. These traits are present in the studied mixtures and combined at different proportions. Combinations of traits in ecosystems subject to fires lead to different fire-related strategies of the species (see Resco de Dios^49^). The results do not show improvements in fire resistance when combining strategies, meaning that stand level mortality did not decrease. In fact, the observed damage was higher when fire strategies were combined. In this case, the combination of strategies could reduce stand stability or resistance when compared to pure stands, especially in stands subject to surface fires. In addition, different strategies result in a ladder effect in the vertical forest structure due to higher connectivity between canopy and understory that favours fire spread. Combining strategies focused on persisting after surface fires (fire-resistant) with strategies focused on persisting after crown fires (fire-resilient) can promote higher biomass densities in intermediate layers of the forest structure, and increasing flammable litter in a more complex and heterogeneous forest structure.

## Conclusions

In this study we assess post-fire mortality according to species composition in mixed stands. Mixed forest can present higher post-fire stand damage than pure stands, as combining species with different fire related traits in some cases increases fire damage. Particularly, mixtures of *P. halepensis* and *P. nigra*, *P. nigra* and *P. sylvestris* and *P. nigra* and *Q. faginea* presented higher stand damage than pure stands of these species. On the other hand, the presence of *Q. robur* in *P. pinaster* stands, even in small proportions, presents a lower than expected post-fire mortality, possibly due to the less likely occurrence of high intensity fires in forests from the oceanic climatic zone inhabited by *Q. robur*. These results provide empirical evidence for the role of mixed forests under fire disturbances and can help design fire prevention strategies aiming at increasing stand resistance to fire through long-term changes in species composition. Promoting species and mixtures that can enhance stand resistance is key to prevent and mitigate the negative effects of forest fires, building fire-resistant landscapes that can cope with the projected increase in fire frequency and extent. Our findings can be further explored to provide silvicultural recommendations to enhance fire prevention strategies.

## Methods

### Data sources

Data about tree measurements and fire damage was retrieved from the second (1986–1996) and third (1997–2007) Spanish NFI^50,51^, which is based on a 1 km x 1 km network of over 80,000 permanent plots with records concerning plot and tree-level variables of forest descriptors and damage occurrence. Variables considered for modelling were derived from second-NFI trees presenting fire damage in the third-NFI. The analysis only included plots with measurements in both inventories. Morphological traits from the species present in the plots (listed in Table 2) were obtained from Paula et al.^52^ and Tavsanoglu and Pausas^53^ (BROT database), as well as from Tapias et al.^54^ (for pines).

### Fire damage model

A model was developed to account for variation derived from the stand structural characteristics which are linked to fuel configuration. The response variable was the proportion of dead trees due to fire in a stand, derived from the NFI variables depicting tree status (dead or alive), type of damage and its importance. The model used a weighted generalised linear approach with the logit of the proportion of dead trees due to fire per plot as the link function and the number of trees per plot as weights. Depending upon the residual deviance and the degrees of freedom of the resulting model, the residuals were assumed to follow a quasibinomial distribution. Variables considered were related to stand structure and topography, and included predictors or variable interactions already analysed in previous studies with similar model approaches in the region^23,35^. Variables not statistically significant, with weak influence on the model, or not being geographically consistent across all studied stands were disregarded. For example, altitude was not significant when combined with other variables, and some combinations of diameter variation with regards to the mean diameter (Sd x Dq^*−*1^) showed important spatial aggregations and opposite coefficient values when modelled in particular regions, indicating regional differences in the model response. The variables included in the final model were: *slope* (stand slope, classified in five categories: < 3%, 3–12%, 12–20%, 20–35%, > 35%), stand *basal area* (G, in m^2^ ha^*−*1^), and *basal area* divided by the *quadratic mean diameter* (G x Dq^*−*1^, which is non-linearly related to number of trees per ha) following:

1 $$logit (y) = {\beta }_{0}+ {\beta }_{1}G+{\beta }_{2-5}Slope+{\beta }_{6}G {Dq}^{-1}+\varepsilon$$

Variance Inflation Factors were used to test potential correlations between the modelling variables.

### Species mixtures and fire-related strategies

The main assumption was that the model accounted for most of the variation in fire damage attributed to stand structure; this allowed us to explore the model residuals with regards to the species composition of each forest. For that, the available plots were classified according to their composition, in pure stands (100% of the stand basal area from a single species) or mixed forests (with three mixture degrees, 25%, 50% and 75% of the basal area from a single species). Tree species represented in fewer than 20% of the plots were disregarded (41 species). The remaining species were predominantly pines and oaks. Stands with more than two species with a relevant amount of basal area were uncommon and were removed from the calculations (i.e. 15 plots with several pines and oaks mixed, each occupying more than 20% of the stand basal area). Finally, based on their representativity, and trait data availability, 6 mixtures with 8 different species were considered for the mixtures analyses (1451 plots).

Once the main mixtures were defined, the species were classified according to their fire-related traits into different strategies or responses to fire. The considered traits were: resprouting capacity after fire, bark thickness (in mm), post-fire seedling emergence, serotiny (% of serotinous cones), cone persistence (in years) and self-pruning capacity of dead branches. These traits are mostly related to fire resistance, post-fire regeneration and survival of tree species, but also to their role in the vertical and horizontal spread of fire. Depending on the combination of traits, species were classified as: *fire-avoider*, *fire-resistant* (species that do not resprout and do not have fire stimulated recruitment but do have thick barks and self-pruning ability) or *fire-resilient* (species that resprout and/or rely on post-fire stimulated recruitment). This classification was based on existing classifications including fire-stimulated recruitment and resprouting ability of the species^55^, as well as classifications related to the influence of the analysed traits on fire behaviour and resulting damage^40,42^.

The classification of species according to their fire-related traits (Table 2) resulted in two different strategies: *fire-resistant* and *fire-resilient*. There were no species that could be classified as *fire-avoiders* in the analysed plots: *Pinus uncinata* would fall within that category, but it was excluded from the analyses due to its low occurrence in burnt plots. Fire-resistant species, like *P. nigra* and *P. sylvestris*, protect their tissues from heat behind their barks and by self-pruning their branches to prevent surface fire from spreading to the canopy. *Pinus pinea*, also a fire-resistant species, was not common in the burnt plots. *P. halepensis* and *P. pinaster* were classified as fire-resilient species, as these rely on the long-term storage of seeds and their post-fire emergence to re-colonise burnt areas. *Quercus* or Oak species were difficult to classify, as they share traits from both resistant and resilient strategies. For example, we could classify them as fire-resistant due to their thick bark, which allows them to resist low-intensity surface fires, but could also be considered fire-resilient species as these also persist after high-intensity crown fires. Based on the resprouting capacity, the final decision was to classify them as fire-resilient. In cases where species presented traits from two different strategies, the most determinant traits to choose a strategy were: resprouting ability and post-fire stimulated recruitment.

**Table 2:** Fire-related traits^a,b^ and fire strategy for the main species analysed.

Species	Resprouting capacity	Bark thickness, mm	Post-fire seedling emergence	Serotinous cones (%)	Cone persistence, years	Self-pruning	Fire strategy
*P. sylvestris*	no	24 (32)^a^	low	0	1–3	yes	Fire-resistant
*P. nigra*	no	31 (34)^a^	low	0	1–3	yes	Fire-resistant
*P. pinaster*	no	35 (30)^a^	low	2–82	2–40	yes	Fire-resilient
*P. halepensis*	no	30 (30)^a^	yes	40–80	5–20	no	Fire-resilient
*Q. ilex*	yes		no				Fire-resilient
*Q. faginea*	yes	thick	no				Fire-resilient
*Q. pyrenaica*	yes	moderate	no				Fire-resilient
*Q. robur*	yes						Fire-resilient

In parenthesis, mean tree diameter at breast height.

*Sources* ^a^Tapias et al. 2004, ^b^BROT database.

Then, these fire strategies were linked to the different mixtures, aiming to understand whether the combination of different strategies had an influence on stand damage, which was statistically tested using non-parametric Kruskal–Wallis tests and post-hoc Dunn´s tests performed using the R packages dunn.test^56^ and stats^57^. Therefore, two types of mixtures were analysed, mixtures of species and mixtures of fire-related strategies.

### Supplementary Information

Supplementary Information.

## Data Availability

The datasets analysed during the current study concerning fire damage and forest stands are publicly available from the Spanish National Forest Inventory (Ministerio para la transición ecológica y el reto demográfico, www.miteco.gob.es). Data concerning traits is available in the BROT database (https://doi.org/10.1038/sdata.2018.135).

## Abbreviation

NFI: National forest inventory
