# Supplementary material for: Impact of species composition on fire-induced stand damage in Spanish forests
Source: Sci Rep. 2024 Apr 13;14:8594. doi: 10.1038/s41598-024-59210-4 (PMC11016083; doi:10.1038/s41598-024-59210-4)
Supplement: Supplementary file 1 — Supplementary Information. [file 41598_2024_59210_MOESM1_ESM.pdf]

SUPPLEMENTARY MATERIALS

Table A1. Stand structural characteristics in the analysed mixed plots. Mean values are represented for each variable. Three mixture levels are considered (1: 75%, 2: 50% and 3: 25).

Species	Mixture level	Stand basal area ($m^2\ ha^{-1}$)	G x Dq^{-1}	Standard deviation of tree diameters (cm)	Top height understory (dm)	Fire damage
P.halepensis - Q.ilex	1	10.17	0.62	5.34	5.57	0.51
	2	5.43	0.35	5.28	6.36	0.41
	3	6.24	0.57	3.41	5.87	0.63
P.halepensis - P.nigra	1	8.23	0.46	4.63	5.22	0.53
	2	9.98	0.64	4.11	5.5	0.64
	3	8.81	0.58	4.29	5.42	0.47
P.nigra - Q.faginea	1	4.58	0.38	4.56	5.57	0.59
	2	6.19	0.44	3.66	5.28	0.77
	3	11.44	0.75	4.86	5.65	0.57
P.nigra - P.sylvestris	1	14.23	0.96	4.6	6.12	0.57
	2	13.12	0.93	3.92	4.56	0.63
	3	14.76	0.77	4.84	5.1	0.38
P.pinaster - Q.robur	1	9.06	0.35	7.96	12.2	0.11
	2	4.93	0.23	7.59	7.11	0.06
	3	8.34	0.4	7.09	13.4	0.01
Q.robur - Q.pyrenaica	1	14.97	0.46	8.97	5.27	0.05
	2	9.34	0.42	8.85	7.37	0.11
	3	8.07	0.36	7.87	16.5	0.12
